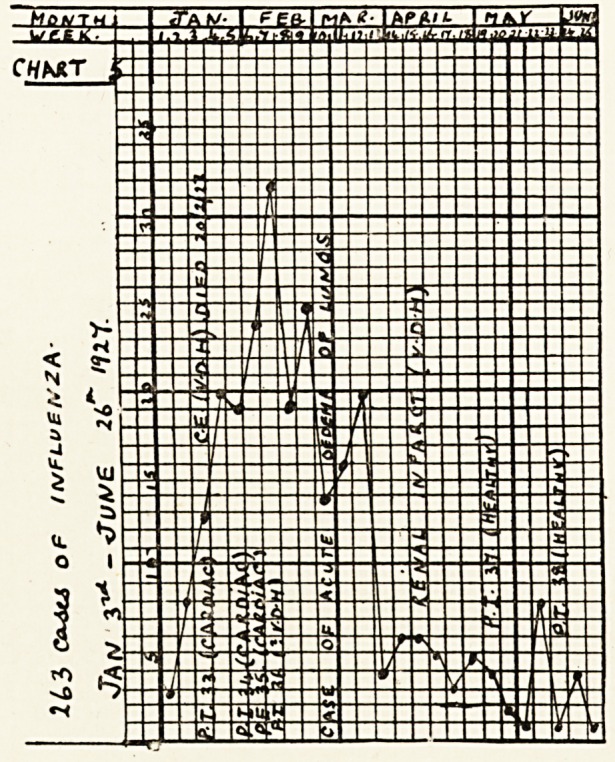# The Infective Factor in Thrombosis and Embolism

**Published:** 1930

**Authors:** Ambrose W. Owen

**Affiliations:** Honorary Surgeon to the Aberdare General Hospital


### THE INFECTIVE FACTOR IN THROMBOSIS AND EMBOLISM.

BY

AMBROSE W. OWEN, M.D.,
*Honorary Surgeon to the Aberdare General Hospital.*

---

THE subject of embolism may not appear at first to be one of great interest to the general practitioner. I say this because it is rarely that one hears the words embolism and infarction in discussions between friends in general practice. I venture, however, to suggest that it is a subject of great practical importance, on account of the common occurrence of embolism in general practice. My attention was first drawn to the matter during an investigation, which has now extended over nine years, into all the cases of influenza (now numbering well over 2,000) which I have visited in their homes. I found that the first case of cerebral embolism which I saw in the period mentioned occurred as a complication of a definite attack of influenza in the March, 1919, epidemic ; this fact induced me to analyse other cases of embolism, and a conclusion I could not resist was that influenza was one of the most important predisposing causes of embolism. Moreover, on account, latterly, of the increasing prevalence of influenza—a disease which has appeared in epidemic form almost every year since the outbreaks of 1918—embolism, which one looked upon not so many years ago as a clinical rarity, has become a common phenomenon in one's practice. The impressions on the subject which first occurred to me in 1919 have been strikingly strengthened since August, 1925, since when I have had an exceptional number of cases of influenza and acute tonsillitis. It will be seen that many of the cases here described were seen in this latter period; I have intentionally chosen them as being most fresh in my memory.

It is my main purpose, indeed, in this essay to direct the attention of the reader to the occurrence of embolism in the familiar motley which we call "influenza," and I hope to produce sufficient evidence to show that *any* variety of embolism may occur during or after influenzal attacks. Embolism, for the most part, has been associated in the text-books with injuries, operations, and disease of the heart valves. It is true that venous thrombosis is described in all books as a possible complication of any of the infectious diseases, and that embolism may therefore be a rare complication of any of the latter; it is true that in pyæmia we have a recognized series of embolic processes. Yet we have so great an authority as Lockhart-Mummery[1] making the following statement: "There is no proof that the presence of septic organisms, or their toxins, in the blood induces intravascular clotting; and, on the other hand, septic diseases which are not the result of wounds, such as whitlows and abscesses, sapræmia, scarlet fever, etc., are not complicated by emboli and infarcts." I hope to prove this statement certainly not true for influenza and acute tonsillitis, both of which I suppose can be put in the category of septic diseases.

The fact, also, that I have not seen in any description of influenza or tonsillitis any stress laid on these embolic phenomena, is an additional reason for broaching this subject now. I believe that the symptoms in many obscure cases that puzzle us are often of an embolic origin ; reference will be made to such cases in the paragraphs that follow.

Perhaps the most useful way of discussing the subject will be by considering the more important types of embolism separately.

*Pulmonary Embolism.*—This is the most familiar type of embolism, as it is generally the easiest to diagnose. It is the type we read of most frequently. It occurs after injuries of the long-bones and severe contusions ; there are the sudden deaths from this cause after abdominal and pelvic operations and after child-birth, tragic examples of which most of us can call to mind. These cases are explained as being due to the formation of a clot in the veins of the pelvis or lower limbs, which empty direct into the right heart, and thus have direct access to the pulmonary artery. The apparently anomalous fact that operations on the gastro-intestinal tract—the veins of which open into the portal vein—are also sometimes followed by pulmonary embolism, is explained by some as also being due to clotting in the veins of the pelvis and lower limbs, the clotting in these cases being said to be due to the prolonged immobilization in bed to which these operation cases are necessarily subjected.

There is no doubt that in the past the tragic suddenness of the fatal event in many of these cases has distracted our attention from the minor or non-fatal cases, which are certainly more common than the fatal cases, and we have been apt to forget that there are *degrees of embolism* which depend upon the size (and nature) of the clot, and therefore on the amount of pulmonary circulation obstructed. This fact was emphasized at two recent discussions on post-operative pulmonary embolism, at which able papers were read by Lockhart-Mummery [1] and E. C. Lindsay. [2] Wharton and Pierson [3] had previously expressed it as their opinion that 40 per cent. of cases diagnosed as pleurisy and 12 per cent. of cases diagnosed as pneumonia after operation are in reality cases of pulmonary infarction and embolism. Curiously enough, I came to a very similar conclusion long before I had read of these observers' work, namely, many of the cases which we diagnose as influenzal pleurisy and influenzal pneumonia are really examples of pulmonary infarction, and a certain proportion of the sudden deaths which occur after influenza are due to massive pulmonary embolism. Unfortunately, post-mortems are difficult to obtain in general practice. It took me many years to realize these facts, but I was persuaded of them by comparing frank cases of pulmonary infarction, such as one sees after injury and abdominal operation, with the type of case so often seen in influenza. I have in mind, as I write, three cases seen in the last few years. The first was a fracture of both leg-bones, the second a severe crush of the ankle. Both these cases, about ten days after receipt of the injury, developed an agonizing pain in the chest accompanied by cough, fever, dyspnœa and cyanosis ; rusty sputum was present, and the physical signs were those of a fairly extensive pleurisy with no suspicion of bronchial breathing. In these cases one naturally thought of embolism, and the physical signs were those of infarction. The third case, three weeks after operation on a septic gall-bladder, developed slight fever and cough, which were associated with a pleural rub, spat up a little blood, but got well in a few days. Here again "septic embolism" was naturally suggested. Now these cases resemble exactly the "atypical" cases of pneumonia seen during influenza.

Let us take a common type of case which we diagnose as influenzal pneumonia. The patient, perhaps a few days after he seems to have recovered from an attack of influenza or tonsillitis, has an acute pain in the chest, is dyspnœic and cyanosed, spits up bloody sputum, and on examination there is fever, a pleural rub, suppression of the breath sounds and some dulness. We say to ourselves: "There is no bronchial breathing, but I suppose it must be pneumonia." Often the temperature is not very high—perhaps no more than 101° F.—and may be normal on the third day, so that we begin again to doubt our diagnosis, and console ourselves by calling the case a "pneumonia of short duration." This type of case, I submit, is a typical example of a pulmonary infarction. The mechanical, as opposed to the inflammatory nature of the case, is seen in the fact that the patient may be out walking on the tenth day! Another important point is that the sputum, which I am afraid the doctor does not always ask to see, is quite different from that of pneumonia, and is generally brought up in separate blood-sputa-tenacious lumps of a reddish-brown colour, reminiscent, according to the degree of discoloration present, of plum, gooseberry or strawberry jam. In some cases the lumps of sputum floating in the fluid used to receive it remind one of a grape, the contents of which have been squeezed out of the skin; I have heard a patient say that she spat up "something like a grape." I will henceforth describe this sputum as jam-sputum—the most appropriate name I can think of to describe it.

In some cases of influenza there may be no signs at all in the chest, in others there may be signs of bronchitis. In both cases jam-sputum may be spat up, showing that an infarction is present. This type of case is often mistaken for phthisis, and may be sent to a tuberculosis expert and even find its way to a sanatorium. Far be it from me to discourage investigation into this type of case, but I am sure many patients have been unnecessarily frightened in this way. I have made the mistake in several cases myself when less experienced ; not a single one of the cases—and I have followed many since 1918—has developed phthisis. In May, 1926, a servant girl suffering from an attack of influenza when home on her holiday, returned to her situation in London before she was properly fit. Shortly after her arrival there she spat up what she described as some " clots of blood." Her mistress took her to a doctor who diagnosed phthisis, and ordered the girl home. As I had seen about this time a fair number of cases of influenza with jam-sputum, I put this girl in the same category. I, however, sent her to an expert, who failed to find any evidence of phthisis. Seen in December, the girl looked hale and hearty.

Both the two previous types of case I believe to be due to small pulmonary infarctions deep down in the lung, and thus causing no definite sign, other than the sputum, of their presence.

The following are the histories of some interesting and typical cases of pulmonary infarction, all of them recent and still fresh in my memory :—

**Case No. 1.**—G. I. T., aged 37, was seen on 3rd April, 1926. Diagnosis : Influenza and tonsillitis. Temp. 104° F. No cough nor respiratory symptoms. He got well in a few days, but a relapse occurred on the 8th. There was now severe bronchitis and cyanosis. No pleural rub nor bronchial breathing heard. Later jam-sputum expectorated. He developed jaundice, but made a good recovery. No evident heart disease. Seen in August, he said he was very fit. In 1927 he got what looked very much like a coronary infarction.

**Case No. 2.**—W. R. G., aged 45, a robust man with a sound heart, was first seen with influenza and tonsillitis and a temperature of 103°F. on 18th February, 1926. No cough. His temperature came down, he was much better in every way, and he got up on the 20th. I did not see him on the 21st. On the 22nd he told me he had been " in agony all night " with a pain in the right chest. There was now severe cough, dyspnœa and cyanosis. The two latter were out of proportion to the pulse and temperature, which were 84 and 99° F. respectively. There was extensive pleural crepitus and suppressed breathing over the front of the right lower chest. I diagnosed infarct. The next day there was typical jam-sputum. The temperature was now normal, pulse 80. An enormous amount of the hæmorrhagic sputum of an unusually sticky mucinous character was voided, followed later by much black (coal dust) sputum ; the latter was evidence of destruction of pulmonary tissue. The sputum was examined pathologically in London, and it was reported that the sputum was undoubtedly from a case of pulmonary infarction, and contained pneumococci, streptococci, and a bacillus resembling B. influenzæ. It is interesting to note that this man, like Case No. 1, also developed jaundice for a few days—evidence, I suppose, of a hæmolytic process. The patient, who was rather fat, lost over three stone during his illness. He took a long time to pick up. The prognosis with so large an infarct seemed doubtful. Seen in December, he looked very well, and an X-ray showed an area of fibrosis in the right lung.

**Case No. 3.**—F. S., aged 43, was first seen on 4th December, 1925, with a septic throat Three other members of his family were similarly affected during the same week, the condition being epidemic here at the time The man was always unhealthy-looking, but had no definite signs of heart disease that I could detect The patient got much better, and had got up and walked about. On the 13th he complained of an acute pain in the left chest, and on the 14th there was a pleural rub and jam-sputum. When he seemed to be recovering he complained of the same thing on the right chest, where a pleural rub was also heard. The temperature never went above 100° F., was subnormal for the most part, and after the development of a typhoid state some effusion in the right chest was found to be sero-purulent. I considered operation out of the question—we were dealing with a pyæmic condition—and the patient died on 24th December. As there was a history of phthisis in the family, the sputum was examined for tubercle bacilli, but was negative. The condition of the lungs was evidently secondary to the throat condition.

**Case No. 4.**—(Pulmonary infarction is not an uncommon condition during pregnancy.) Mrs. P., aged 32, seven months pregnant, was seen on 10th May, 1926. She was breathless and cyanosed, but on examination the temperature was only 99·6° F., and the physical signs in the chest were indefinite. As there was a good deal of influenza about at the time, I was inclined to look upon the case as a deep-seated influenzal pneumonia, but on the fourth day of the illness I observed the typical jam-sputum, and on the seventh day her condition was rapidly approaching normal. There was no sign of phlebitis in the lower limbs. The patient went to term, and was confined on 5th July. It is interesting to note that during the puerperium I discovered a small fibroid to the left of the uterus. Possibly some clot was detached from here (see Case No. 6). Seen in December, the woman seemed her old healthy self.

The two following cases were almost certainly examples of fatal pulmonary embolism during the puerperium :—

**Case No. 5.**—On 4th July, 1921, a healthy woman aged 21, a primipara, died immediately after the third stage of labour was completed. I thought at the time that I was dealing with a case of acute œdema of the lungs. Looking back, however, I am inclined to think that the cause of death was a massive pulmonary embolism, and that the history of a " cold " followed by neuritis a few weeks previously gave the clue to the original cause—influenza. It was certainly curious that I saw, three days later in the same street, a young man with an obvious pulmonary infarction.

**Case No. 6.**—On 30th August, 1925, a healthy woman aged 39, whom I had attended for an influenzal cold fourteen days before labour, died suddenly on the ninth day of an aseptic puerperium. Some small uterine fibroids were diagnosed after the third stage of labour. The only complaint during the puerperium were some vague pains in the pelvis. At the autopsy (abdomen only) the fibroids were seen and clotting had taken place in the pelvic veins. Here probably the fibroids, as well as the influenza, took a part in the fatal event, which must have been due to pulmonary embolism. A week later a young man on whom I had operated for hernia had a typical small pulmonary infarction, whilst a few days later I saw two cases of cerebral infarction (see later).

I believe that yet another type of pulmonary embolism takes place during influenza. Most general practitioners can remember urgent calls to patients during the influenza epidemics, the symptoms being a sudden pain in the chest accompanied by a sense of impending suffocation. As I happen to live in a congested area, very accessible to most of my patients, I generally have managed to arrive in time to observe the dyspnœa, cyanosis, and sometimes slight fever which are characteristic of these cases ; it is probable that if my visits had been less prompt the dyspnœa and cyanosis—which pass off quickly—would never have been observed, and I might have dubbed the relatives " fussy " for sending for me in such a hurry. In several of the earlier cases which I saw I made a diagnosis of " acute cardiac dilatation "—on very little evidence, as the patients were mostly healthy. I am of opinion that the symptoms in these cases are due to pulmonary embolism of a fleeting kind. Perhaps the clot is of a soft nature that breaks up easily ; at any rate, the symptoms resemble exactly those which I

once observed when resident in a large hospital, in a patient after an operation for hernia, and which we all agreed were embolic in origin. No definite signs other than those of pulmonary œdema are observed in these cases, though a rise of temperature for a few days after is common, while in some cases the sputum is hæmorrhagic. Probably some of the cases of "acute œdema of the lungs," of which there has been much talk in these recent influenzal years, are of a similar nature. It is only a matter of degree, according to the size and nature of the infarct. If the clot is a large one death takes place, and I have no doubt that some of the sudden deaths after influenza are due to this cause. I have no post-mortem evidence to support my views, and it would be interesting to hear whether the pathologists see these fatal cases of pulmonary infarction—as I believe they must—more often during the influenza seasons.

It is a well-known fact that a non-fatal pulmonary embolism may be followed later by a fatal one (see Case No. 31), and that a pulmonary embolism may be followed by a systemic embolism. Sometimes the pulmonary embolism is preceded by a systemic embolism, and as the condition arising appears anomalous, the condition is called one of paradoxical embolism. I have observed this latter phenomenon on several occasions, and will have a few remarks to make on the subject later.

*Systemic Embolism.* — Under this heading are included emboli carried through or from the left heart and arrested in one of the systemic vessels. These events are seen classically in cases of malignant endocarditis ; here the occurrence of the embolism may be the first clue to the real nature of the case. Many of the systemic embolisms which one observes during influenza, however, occur in patients with apparently sound hearts, and I think a certain proportion of the emboli must come from the pulmonary veins in such cases ; I will refer to both these points later. As stated before, the systemic embolism may precede or follow a pulmonary embolism in the same patient. Moreover, the systemic embolism may occur after injury or operation without the occurrence at all of a pulmonary embolism. To explain such an event, certain observers are at pains to explain that the clot, in order to get from the right to the left heart without affecting the lungs, must pass through a patent foramen ovale. I suspect that there is no need for this ingenious explanation, unless and until it can be conclusively proved that the embolus is derived from the site of operation or injury in all cases.

I will now give a short history of each of the cases whose symptoms I have attributed to systemic embolisms during the period of my investigation.

*Cerebral Embolism.*—It has been a frequently recurring experience of mine to observe that cases which clinically show the features of cerebral thrombosis occur most often during the influenza seasons, and it is my belief that influenza often takes its toll of the old and arteriosclerotic in this way. The features, however, are not very distinctive, and the diagnosis may be open to doubt. Cases of cerebral embolism, however, on account of their more sudden onset and the development of focal signs, are more easy to classify. I have collected during the period nine cases in which the clinical signs were fairly definite of an intracranial embolism, though I am well aware that it may be asserted that the symptoms were due to encephalitic or meningeal changes. With the analogy of the pulmonary infarction in mind, however, I prefer to look upon these cases as embolic.

**Case No. 7.**—A woman aged 56, suffering from rheumatic endocarditis since girlhood, had an attack of influenza and bronchitis during the March, 1919, epidemic. On 14th March she was taken suddenly with a convulsive attack which was followed by aphasia and right-arm monoplegia. Both the latter improved, and the patient got about and lived for four years afterwards.

**Case No. 8.**—A woman aged 36, healthy, developed aphasia and monoplegia on the fifth day of the puerperium. The latter was afebrile, and no source for the embolus could be found. Practically complete recovery. No association with influenza nor any other illness could be demonstrated.

**Case No. 9.**—A man aged 53, an old case of " V.D.H.," was labelled (five weeks before the embolism) " cardiac plus influenza." The hemiplegia came on suddenly, the condition improved considerably, but he died water-logged a month after the embolism.

**Case No. 10.**—A woman aged 52 with high blood pressure and a perforated nasal septum. A convulsion was followed by aphasia and monoplegia during an attack of influenza in the February, 1924, epidemic. Owing to the intensity of the onset and the general condition of the patient, I gave a bad prognosis, but the patient is still (December, 1929) alive, and her only complaint is numbness in the thumb.

**Case No. 11.**—A rather delicate-looking woman aged 41, but with no actual disease history, had an attack of influenza complicated by aphasia and monoplegia in the February, 1924, epidemic. The onset here was rather slower than in the other cases. There is still some weakness of the right grip, and the patient says she cannot pronounce her Welsh words as before. The improvement, nevertheless, was marked.

**Case No. 12.**—A woman aged 28, with choreic endocarditis of many years' duration, had a slight cerebral embolism (convulsive attack) on 9th May, 1925. She had not had influenza, though the latter was very prevalent at the time.

**Case No. 13.**—A woman aged 60, who appeared to show signs of heart failure for two months previously, and whom I had looked upon as a case of " myocarditis," fell down in a convulsive attack on 12th September, 1925 ; she was unconscious only a short while, but eventually all the signs of a cortical hemiplegia developed, and the patient died on 3rd February, 1926. Influenza was prevalent at the time of the occurrence of this stroke, but I could get no history of an attack in her case.

**Case No. 14.**—A woman aged 30, a case of old rheumatic endocarditis since 1910, was seen with severe respiratory influenza in May, 1925 ; influenza of a severe type was epidemic here at the time. Though she had not been " strong " since her rheumatic fever, she had married and borne three children, and had had no definite illness beyond what I had diagnosed as a " septic abortion " in 1921 (though

I have wondered since if her endocarditis was not the cause of the fever at this time). She never recovered from her attack of influenza, became very anæmic, and some tenderness and swelling over the spleen were probably due to a splenic infarct. I had her removed to hospital, but she got no better, and on the very day that I showed her to a medical friend as an instance of a simple endocarditis made malignant by influenza (17th September, 1925) she was taken with generalized convulsions followed by unconsciousness and total hemiplegia, and death in twelve hours. This was a classical case in a patient with malignant endocarditis.

**Case No. 15.**—This was probably a case of cerebellar embolism. A girl aged 16, whom I had attended for chorea and endocarditis in 1922, had enjoyed fairly good health for over two years, and had worked as a housemaid during that period. On 11th November, 1924, I was urgently called at 5 p.m. to see her, and found her very breathless and cyanosed. The mother said she had had a cold for a few days. My diagnosis was " acute cardiac dilatation." The patient rallied, but at 9 p.m., in response to another urgent call, I found her in tetanic-like convulsions associated with opisthotonos—the cerebellar fits described in the text-books as associated with cerebellar lesions. The girl died five hours later. As I was attending at the time a large number of cases of influenza of a fairly severe type, I cannot help thinking that the " cold " from which the girl had suffered was influenza. The obvious diagnosis was " cerebellar embolism," and it is quite possible that the symptoms which I had put down to cardiac dilatation were due to a pulmonary embolism.

It will be seen that, of the nine cases related, an association with influenza was definite in Cases Nos. 7, 9, 10, 11 and 14, whilst influenza was prevalent at the time of occurrence of Cases Nos. 12, 13 and 15.

I do not propose, in this connection, to enter into such a controversial subject as to whether the so-called encephalitis lethargica is a post-influenzal condition. My very limited experience inclines me to the belief that it is. The most typical case of encephalitis of the choreiform variety which I have seen occurred at the height of the February—March, 1924, epidemic ; in another case, also of the choreiform variety, a medical friend of mine had suffered from an influenzal cold ten days before. The latter case—fortunately not fatal—was verified by a physician who has had a fair experience of the disease ; influenza was again very prevalent at the time of occurrence of this case. I will content myself with asking others more qualified to judge if the symptoms of encephalitis lethargica would not fit in with a condition of thrombosis and embolism of the arteries of the basal ganglia. These are end-arteries, and this is the part of the brain most frequently affected.

*Renal Embolism.*—I have long been of opinion that many of the cases which we diagnose in general practice as "nephritis" — whether they be of the so-called *exudative* or *hæmorrhagic* types — are due to renal infarction. I have noticed recently that many other observers are of the same opinion, a fact which only occurred to me when I read the recent interesting article by Wyllie and Moncrieff [4] on "Acute hæmorrhagic nephritis in children." These observers draw attention to the well-recognized fact that the condition is often consecutive to an attack of "acute tonsillitis," and suggest, very rightly, I think, that it is due to renal embolism. I have seen many such cases follow on non-diphtheritic and non-scarlatinal sore throats in children (I saw at least three in the last quarter of 1925), but I have also seen the same condition follow on attacks of influenza in adults, so that the condition should not be labelled a special disease of childhood.

Now, renal infarction, it is well known, may occur in two forms. In the first blood is present in the urine in such quantity as to warrant the use of the labels "hæmaturia" or "hæmorrhagic nephritis;" in the second the clinical features may be those of an "exudative nephritis"—albumen being obviously present, whereas a microscopical examination may be needed to detect the presence of red-blood cells. To put it tersely in Sir Thomas Horder's words, "intermittent albuminuria should, therefore, suggest renal infarction just as intermittent hæmaturia does." [5]

Both types of cases are seen after influenza, as will be seen later. Moreover, as it is natural to expect if we believe the cases have an influenzal origin, one sees in practice little epidemics of nephritis which occur either during or immediately following the epidemics of what one has been in the habit of calling " tonsillar influenza."

As I am revising this section, I notice in the *British Medical Journal* of 11th December, 1926, four cases of " Acute hæmorrhagic nephritis following Influenza," reported by Cook. One of the cases was an adult. Whether one calls the cases influenzal or not makes no difference to their pathology ; they are obviously of an infective origin. One rarely sees the old-fashioned type of " Bright's disease " in practice nowadays ; most of the cases of nephritis seen are of the nature described above. The important characteristic of the cases—whether occurring in adults or children—is the excellent prognosis. I can remember but one case (No. 17) which failed to clear up promptly.

There is also a type of case following influenza in which the features are those of a pyelitis. It is probable, too, that these are the result of embolism with sepsis added. I am not confusing B. coli pyelitis with influenza, as the reader may be inclined to think. Whatever may be the predominating organism (and unfortunately one has not the time to classify these cases bacteriologically), there is here, also, a definite preceding sore throat with fever ; moreover, the cases are seen generally during the influenza epidemics. The administration of potassium citrate leads to a speedy cure as a rule, and only rarely have I had to resort to vaccines.

All the cases described above occur during apparent convalescence from the influenza—after the patients are up and about ; it is quite possible that, if I had not been in the habit of making careful notes, in many of the cases, the original illness might have been forgotten both by the patients and myself.

The embolic theory advanced as to the causation of the nephritis certainly receives support from the occurrence, in the same patient, of an obvious embolism in another organ, as in Cases Nos. 18 and 19.

I will now relate the histories of a few typical cases :—

**Case No. 16.**—G. T., male, aged 18, first seen on 24th May, 1926, for influenza, *i.e.* sore throat and fever. When convalescent he came to see me on the 31st, complaining of abdominal pain, and I noticed puffiness of the eyes. The urine contained a fair amount of albumen and red-blood cells microscopically. The patient was not particularly ill, would not stay in bed nor diet himself, but the urine was normal on 30th June, and the boy soon got well. He was previously healthy. This is an example of an uncomplicated case of the exudative type.

**Case No. 17.**—E. R. E., male, aged 45, was seen in my surgery on 24th December, 1925. Diagnosis : Influenza with sore throat and fever. I sent him home to bed, he had got better and walked about, but on 13th January, 1926, he came to see me complaining of swelling of his eyes and ankles, and brought his smoky urine for me to see. He was obviously suffering from nephritis. The urine contained a fair amount of albumen and blood. This man—a hard-working miner—has a dilated aorta, and possibly an atheromatous patch here may have furnished an embolus. The fact remains that there was a definitely observed infective factor in the attack of influenza preceding the nephritis. This man has been under observation for nearly a year, and the renal condition is not yet satisfactory. The man, however, was never of a robust type. This is the only case which I can remember that has not cleared up promptly. It is an example of the hæmorrhagic type in an adult. (1930.—The urine has been quite free for some time and he is working as a miner.)

**Case No. 18.**—This case is of unusual interest, inasmuch as a pulmonary infarction was observed after the development of the nephritis. Mrs. P., aged 39, and always healthy, was first seen with influenza on 29th October, 1925. She improved, but on 16th November I was asked to visit her again, and found she was suffering from " post-influenzal nephritis." On 22nd November, when the renal condition had much improved, I received an urgent message to see her, and found her in an alarming condition of dyspnœa and cyanosis. She complained of an agonizing pain in the chest. A well-marked pleural rub was heard on the 23rd, and on the 24th and following days she spat up very characteristic blood-sputa, which confirmed beyond all doubt our diagnosis of pulmonary infarction. The woman has no heart disease, and is now in good health, though she says she has occasional pain over the region of the trouble last November. About this time I saw no less than six cases of post-influenzal nephritis.

**Case No. 19.**—In this case, also, a pulmonary infarction occurred after the nephritis. G. C., male, aged 28, was first seen on 15th February, 1926. He had the type of influenza with sore throat that was raging at the time (see Case No. 2). On 28th February his ear " burst " as the result of otitis media—a very common complication of influenza nowadays. On 13th March my note was " cardiac sounds soft and muffled ; swelling of face and eyes, which he says he first noticed on the 10th, after he had got up and about." The urine was now loaded with albumen and blood was present. On 20th March the urine was clear and remained so. He appeared at last to be getting better, but on 30th March he had an acute pain in the chest associated with fever and a pleural rub, and on 4th April he spat up the characteristic blood-sputa. He suffered later from a swelling of one hand and arm, which I thought must have been due to a peripheral embolism, but it must have been of a transient nature, as the swelling disappeared. The man was extremely weak and anæmic for some weeks, and I kept the possibility of malignant endocarditis in mind. The man improved, and is now working as a builder's labourer, and says he feels as fit as ever. I rather suspect, however, that his cardiac valves will trouble him at some future date.

If we assume the renal condition to have been embolic in the last two cases, we have here two examples of retrograde embolism.

**Case No. 20.**—H. E., a man aged 51, had a severe attack of influenza complicated by "pleurisy" at the latter end of December, 1920. On January 2nd, 1921, copious hæmaturia was observed. I attributed the hæmaturia to renal infarction, as further investigation failed to find any other cause for the bleeding. Possibly the "pleurisy" was due to pulmonary embolism. At the time I could find no definite evidence of heart disease, though I knew the man was never robust. It is interesting, therefore, to state that nearly six years later (September, 1926) the man began to show signs of cardiac failure, and I have now labelled him "myocarditis." It is curious that at the time of this man's illness I saw four probable cases of pulmonary infarction in twelve days. (see page 54) He is still (1930) not working, very short of breath.

*Mesenteric Embolism.*—The usual text-book description of mesenteric embolism concerns itself with an account of the complete blockage of the superior mesenteric artery—a condition generally occurring in a patient the subject of cardiac valvular disease, leading to gangrene of the intestine, and associated with an appalling mortality even if promptly treated by surgical measures. I am perfectly convinced, however, that just as we get minor degrees of pulmonary embolism, so we sometimes have to deal with minor degrees of mesenteric embolism. Now, there is a fascinating and puzzling group of cases occurring during influenza in which pain, generally in the right iliac fossa, vomiting, often fæculent, distension of the abdomen, and fever, may lead one to suspect acute appendicitis or even intestinal obstruction ; there is, however, something lacking in the clinical entity, we stay our hand and find that the patient recovers without operation, though not without a good deal of abdominal distension and discomfort which may last for weeks, and which I believe are due to abdominal adhesions. These cases do not seem to me to be explained satisfactorily by the term " influenzal typhlitis " often used in this connection. It is my firm belief that the symptoms in these cases are due to a mild degree of mesenteric infarction. The clot may be a soft one that breaks up easily so that its particles pass on, or a small one, so that the circulation gets re-established and thus no gangrene of the intestine occurs; in the meantime, however, the infarction has caused a sufficient degree of ileus (consequent on the intestinal anæmia) to alarm the observer. I take it that the freedom of anastomotic communication varies in different people, and that while the freedom of communication is fairly complete in most mesenteries, end-arteries exist in this situation in some bodies. The freedom of communication of the vessels of the lower end of the large intestine is never as complete as that of the vessels higher up. A small clot may cause only some exudative roughening of a limited portion of intestinal peritoneum with perhaps some localized adhesions and a more or less early return to normal ; a larger infarct may cause a small patch of gangrene which may perforate if it is not covered over with a protective pad of omentum. If the clot is a large one, we get the classical symptoms and signs of thrombosis and embolism of the mesenteric artery—a condition so rarely seen that it would be difficult to ascertain whether the pathologists see it more often during the influenza seasons. While patients with valvular disease are naturally more prone to the above accidents, I fail to see why they cannot, like other varieties of infarction, occur in healthy subjects.

The following cases illustrate the occurrence of peculiar and puzzling abdominal symptoms during influenza :—

**Case No. 21.**—W. T., a man aged 48, with " myocarditis " of some years' duration, was seen with peculiar pulmonary symptoms and signs on 10th March, 1919. Influenza was epidemic at the time, but I did not label him " influenza," as I had the cardiac condition in mind. On 21st March he presented symptoms suggestive of intestinal obstruction. He was an impossible subject for an operation, and he died on 1st April. I was much puzzled by the case at the time, as also was a colleague who saw the case with me. Looking back, however, in the light of subsequent experience, it seems clear that the lung condition was one of infarction, and that the abdominal condition was one of mesenteric embolism. I discovered some time afterwards, whilst engaged in my investigation, that I had attended other cases of embolism during this epidemic. It is difficult at these busy seasons to think coherently. It is the calm survey after which is often so illuminating

**Case No. 22.**—B. J., a healthy woman aged 24, was first seen on 11th February, 1919, at the onset of an influenza epidemic. Severe, protracted bronchitis followed the influenza. The patient went rapidly downhill, with a septicæmic condition, and on 13th March severe abdominal pain was associated with a lump in the abdomen and peritonitis. Operation was declined, and the patient died on the 14th. I was much puzzled by this case, which I had reported to the Medical Officer of Health as a possible case of enteric. The parents—peculiarly stubborn people—had refused to allow me to take a sample of blood. Though all the illness had resembled a severe case of influenza, and though the latter was epidemic at the time, the presence of an abdominal lump had puzzled me, and made me think of perforation of a typhoid ulcer. I believe now that the lump was due to a mesenteric embolism. Horder,[5] in his interesting lectures on endocarditis, describes what appears to be a similar case. The difficulties of diagnosis—even to the expert—are apparent from his remarks that " despite the unusually discreet nature of the lump . . . the case would doubtless have been considered one of acute appendicitis, had it not been that . . . the patient became suspect on the score of a possible septic endocarditis."

**Case No. 23.**—M. D., a robust woman aged 46 (who, however, has a child with congenital syphilis), had an attack of influenza and tonsillitis on 9th April, 1925, and during the attack she developed abdominal symptoms which much puzzled a locum who was helping me at the time. The case resembled a case of acute appendicitis with much distension, but owing to the attack of influenza I thought it a case of embolism, and the patient got well without operation. It is interesting to note that this woman had fractured her tibia on 19th February, about fifty days before her influenza ; possibly a clot was detached from the leg. Why, in this case, however, was the event noticed during the attack of influenza, or had the fracture anything to do with it ? The following July I had occasion to open the abdomen for the performance of a long-contemplated ventral suspension, and took the opportunity afforded of inspecting the appendix, mesentery and intestine. The appendix was normal and free of adhesions, the mesentery was unduly thickened in parts, and the intestines were mottled with greyish-white patches—recent, and evidently the remains of peritonitis. There were also several recent adhesions between various parts of the small intestine. I think that the whole condition was due to the attack of abdominal influenza in May.

The following case, unconnected with influenza, I looked upon as an infective endocarditis with embolic features :—

**Case No. 24.**—H. W., a man aged 38, who had always been healthy, but who had suffered from syphilis in 1918, began to show indefinite signs of failing health in July, 1925. The nearest approach to a diagnosis that I could get was a label of " cardiac dilatation." No murmurs were heard. On 14th September gallop-rhythm was observed. After a period of going downhill he was taken on 31st October with abdominal pain and fæculent vomiting—a condition resembling a subacute obstruction. I came to the conclusion that the case was one of endocarditis of probable specific origin, and that the abdominal symptoms were probably due to small mesenteric emboli. The latter suggestion received support a few days later, as on 2nd November a pain in the chest was accompanied by a pleural rub, on 4th November some bloody sputum was expectorated, and two days later about half an ounce of pure pus was voided. The patient died in a toxic state on 8th November. It would seem that here a pulmonary embolism followed a mesenteric embolism.

There is some support for my theory of a minor degree of mesenteric embolism. Maingot[6] recently reported a case of perforation of the jejunum following what was called a lobar pneumonia, and in which he successfully operated. I was interested to see that he attributed the perforation to a " septic infarct " of the intestine. This case occurred in November, 1924, when influenza was very prevalent. Mr. Maingot has kindly informed me that he has had a similar successful case since ; in this case a perforation occurred in the intestine of a child after an attack of influenzal bronchitis. I also operated on a child in the 1918 epidemic for infarction of the intestine.

With regard to the infarcts being septic, I think that most of the emboli of which one sees the effect during influenza must be of what the pathologists call a bland (aseptic) nature, and sepsis as we usually understand it, *i.e.* the formation of pus, is not necessary for their initiation. Occasionally we get septic infarcts —Cases Nos. 3 and 24 are examples. I saw also in May, 1925, when influenza of a severe type was raging in my practice, a case of pulmonary abscess in a child, which I think was originally due to a central pulmonary embolism, the latter being diagnosed as influenzal pneumonia.

I may add that no melæna was observed in any of the cases described above as probable mesenteric embolisms. It probably needs a large area of intestine to be involved, or the erosion of a large vessel, to cause this symptom. Possibly, some of the melæna which we observed frequently in the November, 1918, epidemic, was caused in this way. The following case seemed to be an instance of an erosion of a large vessel in the intestine by an infarct :—

**Case No. 25.**—W. E., aged 40, had a severe internal hæmorrhage on 13th June, 1925. This was followed by melæna on the 15th. An X-ray of the stomach gave no illumination as to the cause of the trouble ; an exploratory operation on 7th December showed two healed rounded patches in the third part of the duodenum—recent, and evidently the cause of the hæmorrhage in June. They were, I think, healed infarcts. It is possible that many duodenal ulcers originate in this way. This man has a dilated heart with no murmurs—there is probably endocarditis present. He has left the district.

*Splenic Embolism.*—I can remember only one case of splenic embolism associated with influenza, Case No. 14.

*Coronary Embolism.*—It is well known that sudden and complete blockage of a coronary artery is a common cause of sudden death. We were all taught as students the effect of gradual blockage—an area of infarction in the cardiac wall followed by scarring and a condition of fibrous myocarditis. I doubt, however, if we general practitioners have kept the latter process sufficiently in mind. We see very many cases of "weak heart"—many of them following the infections, and our usual label of "myocarditis," though a pretty accurate one, becomes so habitual that we are inclined to forget that here again the process of embolism is the most important in the ætiology. Thanks, however, to the recent work of certain observers, notably Gibson, [7] Coombs and Hadfield, [8] the subject has once again been emphasized to us. Gibson is at pains to point out that what he calls "ischæmic necrosis of the heart muscle" (cardiac infarction) is not only a cause of sudden death and angina pectoris, but is an important cause of chronic heart failure also. He states, indeed, that "chronic cardiac failure in old people in the absence of syphilis, old rheumatic affections of the heart, and pernicious anæmia, is almost certainly due to ischæmic necrosis," and that "chronic cardiac failure plus evidence of aseptic embolism makes the diagnosis of ischæmic necrosis practically certain." I have no hesitation, as a general practitioner relying only on clinical evidence without the support of post-mortem evidence, in fully agreeing with these two sound statements. Case No. 13—the woman with "myocarditis," who developed a cerebral embolism—was a typical example of ischæmic necrosis. There was no sign of rheumatism or syphilis in her case. A more dramatic instance of ischæmic necrosis was the following:—

**Case No. 26.**—M. M., a woman aged 55, had always been exceptionally healthy, and the only attendance I had given her in eight years was for a Colles' fracture. I first saw her on 28th September, 1925, for what I took to be a disordered action of the heart of functional origin, and thought very little of the matter. On 15th October, when I was sent for, I was horrified to find her in bed with œdema up to the waist, and with a large pulmonary infarct. After some slight improvement she fell back dead in bed on 7th November. The duration of the case was thus only forty-one days. I could elicit no history of a cold or influenza, though there was a good deal of the latter about at the time.

We probably see in practice, also, cases of coronary infarction which, though causing alarming symptoms, are not immediately fatal. They are analogous to the cases of pulmonary infarction which I have described on pages 34 and 35. The following is an example:—

**Case No. 27.**—Early this year (1926), D. J., a man aged 67, in failing health since he had an attack of influenza with severe delirium in 1923, came to my surgery gasping for breath, and with severe precordial pain. He had been suffering from a " bad cold " for a few days. He was ashy-pale, and on examination his heart was fluttering, and there was a marked pericardial rub, which latter was probably the outward sign of a cardiac infarct. I sent the patient home to die, as I thought, but, to my surprise, he got better, and is now walking about much improved in health. It would not surprise me, however, if he drops down dead at any time ; it is well known that a patient may live for years after a cardiac infarction.

I had been of opinion, long before I read of Gibson's and Coombs' work, that, just as some of the sudden deaths after influenza are probably due to pulmonary infarction, so also a certain proportion are probably due to coronary infarction. If pulmonary infarction in its minor forms is such a characteristic of influenza—a fact of which there is no shadow of doubt, as the physical signs are near the surface and can be recognized—it is only reasonable to suppose that infarction occurs in other situations as well, and I think I have given sufficient evidence of this already. Owing, however, to other organs than the lung being deeper down and, so to speak, out of our clinical "reach," it is less easy for us to be dogmatic in our interpretation of the symptoms and signs produced, and unfortunately the general practitioner, though often convinced in his own mind, is denied the post-mortem.

It is interesting to note that the youngest of Gibson's patients—a woman of 25—died after an attack of influenza. Death was sudden, and the post-mortem disclosed "thrombosis of the pelvic veins and an embolus of a coronary artery." It is significant, too, that of the three cases described by Coombs one was said to have had influenza and another influenzal pneumonia.

The sudden deaths from coronary embolism which we may assume to occur after influenza, though more prone to occur in the subjects of old heart or arterial trouble, may quite well—by analogy with our other cases of systemic embolism — occur in healthy and robust subjects. I suspect that if post-mortems were the rule in general practice, we would find that coronary embolism is far commoner than we think. The following are but three cases of sudden death after influenza, most probably due to the above cause :—

**Case No. 28.**—W. W., male, aged 48, an exceptionally healthy colliery official, was attended by me for what appeared to be a mild attack of influenza in the severe January–February, 1922, epidemic. He returned to work without consulting me (though I would probably have given my consent), his wife said he had felt quite fit, but he died suddenly in the pit on the day of resumption.

**Case No. 29.**—J. D., aged 64, arteriosclerotic, had an attack of influenza in February, 1925, when influenza was epidemic. He appeared to improve, I had not seen him for a week, but I was urgently sent for on 27th February. After retiring for the night, he had sat up in bed, gasped and become livid, and had died before I arrived, a few moments after being sent for.

**Case No. 30.**—H. J. F., male, aged 55, whom I had known for eight years, and who had consulted me for only trivial complaints, came to see me in May, 1926. He told me he had had " a dose of 'flu which he thought he could cure himself," but had been " aching all over since." I took it to be a case of post-influenzal myalgia—a very common complaint nowadays. A few weeks later there were signs of cardiac dilatation, and it was this label that I used when I signed the man " on his club." Later still there were severe pains in the epigastrium which, at first, I thought might be due to the aspirin which the man had been taking. When, however, jaundice was observed, in spite of the fact that I saw several cases of jaundice at this time, I thought that there might be a local trouble, such as gall-stones, to account for the abdominal symptoms. An X-ray of the stomach and other investigations were of no help. I believe I would have recommended a laparotomy, had it not been that I was rather dubious as to the cardiac condition. Though the man made little progress, he was able to walk about. When I was away on my holiday in August my assistant was sent for in a hurry one morning, but the patient was dead when he arrived. I think that there was no doubt that the case was one of ischæmic necrosis, and that the jaundice was but a sign of a hæmolytic process associated with the influenza from which the patient had evidently suffered (see Cases Nos. 1 and 2). Gibson, in his article, refers to these cases with symptoms referred to the abdomen.

One could quote many similar cases; my death certificate counterfoils show—and probably those of other general practitioners do also—that a majority of my "cardiac" and arteriosclerotic patients have died during the influenza seasons, most of them suddenly.

*Peripheral Embolism.*—I have personally seen no definite example of the lodgment of an embolus in an artery of a limb, though I suspected a transient embolism of this sort in Case No. 19. A friend of mine, in practice near, tells me of a case of his in which during convalescence from influenza a middle-aged patient developed a cerebral embolism which was followed by bilateral gangrene of the legs, most probably the result of blockage by emboli. I saw, a few months ago, several patients convalescent from acute tonsillitis, in whom slightly-raised bluish-red patches appeared over the fronts of the legs. This condition, which appeared to be of the nature of erythema nodosum, is probably due to emboli in the minute vessels of the skin. I noticed that several of these cases were reported in the medical journals at the time.

Owing to its position and characteristic symptoms and signs, the peripheral embolism should be easy to diagnose, and it is of interest to state that, in spite of its superficial position, operations for the removal of the embolus have not been very successful, as, so far as I can find, only one successful case has been reported in this country, and that by Jefferson [9] as recently as April, 1925. Here an embolism of the brachial artery followed an operation for umbilical hernia. No heart disease was detected, and the patient was healthy. In this case an infarction of the lung followed the peripheral embolism. Mr. Jefferson has kindly informed me that there was no history of influenza in his case, but it is interesting to add that influenza was prevalent at the time of the occurrence.

*General considerations.*—It will be noticed that I have described a fair number of cases of embolism (and I have notes of many more) in which a definite attack of influenza was antecedent. If, as I hope, I have proved to the reader's satisfaction that influenza is often the predisposing cause of infarction, it is but natural to suppose that this is sometimes a seasonal phenomenon, and I have found, far too often for the matter to be considered a coincidence, that "runs" of embolism—or embolic cases—are encountered in general practice. It is true that one sees also cases of embolism in which no apparent history of "cold" or influenza is forthcoming, but as such cases occur in close chronological proximity to other manifestly influenzal embolisms, is it unreasonable to suppose that the blood condition—if such there be—which led up to the embolic event might be the only influenzal manifestation—just as, for example, a neuritis may be? One must allow, too, for the short memories of one's patients. What they may look upon as "a bit of a cold," and forget about soon, may, nevertheless, be influenza, and fraught with grave possibilities later. Not so long ago a doctor friend of mine with encephalitis lethargica, forgot all about the "touch of 'flu" which I remember he had casually mentioned to me over the telephone about ten days previous to the onset of the choreiform movements. What, therefore, can one expect of a layman?

Here are three examples of a "run" of cases of my own in which the symptoms were very probably of embolic origin:—

1. During the March, 1919, influenza epidemic I saw:

(*a*) A case of pulmonary infarction in a pregnant woman of 23, who was suffering from influenza on the 9th.

(*b*) A case of influenza with "jam-sputum," on the 12th. This case, a boy aged 16, at the time was suspected of phthisis and attended a clinic. No bacilli were found. Patient alive and well.

(*c*) Case No. 22.—Mesenteric embolism on the 13th.

(*d*) Case No. 7.—Cerebral embolism on the 14th.

(*e*) Case No. 21.—Mesenteric embolism on the 21st. The initial symptoms of this case on the 10th were probably due to pulmonary infarction.

That is, five cases were seen in thirteen days.

2. At the end of 1920 and beginning of 1921 I saw:

(*a*) A healthy woman who, on the sixth day of an aseptic puerperium, was taken suddenly ill with marked dyspnœa and cyanosis on 22nd December, 1920. The initial label was "? heart or embolism."

(*b*) On 23rd December, 1920, I diagnosed a case (a young child) "larval pneumonia."

(*c*) A man aged 30 with a typical pulmonary infarction on 27th December, 1920.

(*d*) A healthy boy aged 15, who during an attack of influenza showed signs of what I diagnosed as "acute cardiac dilatation."

(*e*) Case No. 20, who had hæmaturia on 2nd January, 1921 (following influenza and pleurisy).

Here five cases were seen in twelve days.

3. Between 30th August and 17th September, 1925—a period of nineteen days—I saw:

(*a*) Case No. 6.—Fatal pulmonary embolism, on 30th August.

(*b*) A case of pulmonary infarction after operation on 7th September. I operated on this healthy man, aged 24, (hernia) on 3rd September. I found out afterwards that he had had "a cold" before admission to hospital.

(*c*) Case No. 13.—Cerebral embolism on 12th September.

(*d*) Case No. 14.—Cerebral embolism on 17th September.

I had seen nothing like a case of embolism since the preceding May. In this month (when influenza was epidemic here) I saw Case No. 12 on the 9th (cerebral embolism), a case of influenzal pulmonary infarction on the 31st, and a young boy whom I had diagnosed as a central influenzal pneumonia; a pulmonary abscess followed, and death from septic embolism of the brain; it is practically certain that the case was originally a central pulmonary embolism.

One could give further examples if one wished to become wearisome. I cannot believe that we are dealing with an extraordinary series of coincidences in the events related above.

The charts show the incidence of influenza in my practice from January, 1923, to June, 1927. The

CHART I

103 CASES OF INFLUENZA.

CASE OF ? CEREBELLAR EMBOLISM.

CASE OF ACUTE OEDEMA OF LUNGS

P.I. 24. (TONSILLITIS)

P.I. 25. (TONSILLITIS)

P.I. 26. FOLLOWED POST-INFLUENZAL NEPHRITIS

P.I. 27. (TONSILLITIS) P.E. 28 (POST-OPERATIVE.)

P.I. 29. (PREGNANT)

P.I. 30. (BRONCHITIS)

RENAL INFARCT (V.D.H.)

C.E. (AET. 70)

P.I. 31. (V.D.H.) RENAL INFARCT.

P.E. 32. PUERPERAL

vertical columns are the weekly periods (1 to 52) when my books are made up. Inserted in them are the numbers of *fresh* cases of influenza *visited* weekly—thus forming the curves. Inserted in them also—in the weeks appropriate to their dates of occurrence—are the cases of pulmonary infarction (P. I.), pulmonary embolism (P. E.), cerebral embolism (C. E.), " ? cerebellar embolism," renal infarction, acute œdema of lungs, etc. I think it will be admitted that the charts show that the occurrence of embolism coincides with the prevalence of influenza. Cases of coronary embolism have been excluded, as the diagnosis of this condition is more open to doubt.

A question I think I may be entitled to ask, in view of the foregoing, is: Are the cases of embolism—pulmonary or systemic—which occur after operation, and which have been so freely discussed by surgeons of late years, due to the operation *per se*, or is some other factor at work ? The usually accepted sequence in these cases is something like this: operation—sepsis—

venous thrombosis—embolism. The word sepsis is an unfortunate one in this connection, as it makes one think of pus, and it is well known that embolism may occur even when the wound has healed by first intention and there is no sign of pus elsewhere. Would not the term infection be more desirable, and is it necessary that the infection be carried by the surgeon's hand ? Might not the infection be present in the patient before operation ? I remember well, when resident in a large hospital, a patient with chronic appendicitis dying of pulmonary embolism on the day before that fixed for operation. A pulmonary embolus was found at autopsy, and there was no pus anywhere. Indeed, I would be so bold as to ask, if the post-operative embolism, in many more cases than we imagine, is not merely a coincidence. Operations are always being performed ; some of them must take place when, to use a tentative expression, " embolism is in the air," and we will thus always have a certain proportion of operations followed by embolism. As I have endeavoured to show, embolism is fairly common in healthy persons apart from operation, and Lindsay [2] has drawn attention to Rupp's figures as showing that " the mortality from pulmonary embolism in internal disease without operation worked out at 1·1 per cent., that is, four times as great as that following operation."

I suspect that of all the contributors to the discussion on post-operative pulmonary embolism at the Royal Society of Medicine on 4th February, 1925, Featherstone [10] came nearest the truth when he " attributed great importance to an epidemic, such as of influenza or ordinary colds, in the causation of post-operative pulmonary conditions."

I have already mentioned, when speaking of the seasonal incidence of embolism, a case of pulmonary infarction following operation which occurred in September, 1925, within a few days of the occurrence of other cases of embolism in patients on whom no operation had been performed. During February and March, 1926, I had a large number of cases of influenza, in which the chief features were tonsillitis and a tendency to embolism during apparent convalescence. Several of these cases have been described. One can surely say at such a time that "embolism is about," and assume that the operation case is exposed to risk of embolism at the same season. The following case may be taken as an example :—

**Case No. 31.**—I operated on Mrs. W., a rather obese woman with a fatty heart, for a twisted ovarian cyst on 27th March, 1926. Operation was performed twelve hours after the onset of the symptoms, was attended with no difficulty, and in the ordinary way the prognosis would have been excellent. On account, however, of the patient's heart, and of the embolic cases I had seen at the time, I actually warned the relatives that there was a risk that a "clot of blood" might pass to the lung. Surely enough, on 8th April—twelve days after operation—the patient was taken ill with an acute pain in the chest, and was so cyanosed and pulseless that the sister in charge thought she would surely die. She rallied, however, brought up some blood-stained sputum later, appeared to improve, but on 28th April—a month after operation—she died with similar symptoms referred to the other side of the chest. It is only fair to add that some sepsis had occurred in the wound. I could not, however, dismiss from my mind the other cases of pulmonary infarction I had seen at the time.

There has been some discussion lately as to whether the post-operative embolisms are more common relatively than they used to be. While some observers state that their frequency has not increased, Gordon-Watson[11] is of the opposite opinion, and he shows that at St. Bartholomew's Hospital for the three years before the war the surgical deaths from pulmonary embolism were 0·5 per cent., for three years after the war they were 1·7 per cent., and in the next two years 2·9 per cent. I attribute this to the increased incidence of influenza during the latter periods. Thus, during the three years after the war, my records show that I

visited 243 cases which were labelled influenza, and in the next two years 481 cases. One has only to con the medical journals to note in the last two years an increased reporting of cases of embolism, some of them even after operations for cataract. Even in the newspapers one is constantly reading of the accounts of inquests in which " a clot of blood " somewhere is said to the cause of death.

Though, as I have shown, infection must play a great part in the production of embolism, speculation is still rife as to the exact mechanism by which the latter is brought about. In pulmonary embolism the embolus must be derived from a clot in one of the systemic veins (generally those of the pelvis or lower limbs), in the right heart, or in the pulmonary artery itself ; in systemic embolism the embolus is derived from a clot in the pulmonary veins, in the left heart, or possibly in the aorta. It is only when the thrombus occurs in one of the comparatively superficial veins that it can be detected by examination. Now, though thrombosis in the veins of the lower limbs is such a well-mentioned complication of influenza, it is extraordinary that in not a single case of pulmonary infarction detailed above was I able to detect any sign of phlebitis in the lower limbs. In fact, I can remember only one case of pulmonary infarction where I was able to satisfy myself of the presence of such phlebitis. On the other hand, Whittingdale[12] reports seeing in the twelve months preceding March, 1926, a large number of cases of phlebitis of the lower limbs with a " large proportion of pulmonary infarcts," and in one case " multiple cerebral emboli," and he asks tentatively if other observers have had the same experience. I have no doubt that his cases, occurring in a period I have also found characterized by a marked increase in the number of embolic cases seen, bear an ætiological relationship to the cases I have described, but it is curious that the manifest evidence of thrombosis was missing in my cases. Probably the large veins of the pelvis are the source of the clots in the majority of cases ; this was so in the only case of mine that came to autopsy. In the cases that follow operation Lockhart-Mummery is probably correct when he says that the site of operation is by no means invariably the source of the embolus.

How are we to explain the systemic embolisms ? Those which occur in the subjects of obvious heart disease are easy enough to explain. What of the systemic embolism that occurs in the previously healthy subject ? Is it due, in influenza, for instance, to a recently-acquired acute endocarditis which is not recognizable clinically ? This theory will explain, probably, such cases as Nos. 19, 22 and 3. I am well aware that the recognition of an acute endocarditis is often a very difficult matter, but against this supposition in many of the cases is the fact that the patients get better rapidly and apparently permanently, a history hardly suggestive of an acute or malignant endocarditis. It seems to me—I speak with due humility—that the pulmonary veins must be an important source of the systemic embolisms in healthy patients. I had long conceived such a thrombosis as accounting for the curious physical signs met with in the chests of influenzal patients, before I found that Glynn[13] had placed pulmonary thrombosis on a sound scientific footing. The latter, indeed, goes so far as to say that what we call pulmonary embolism is really primary thrombosis in the pulmonary arteries. I fail to see why thrombosis should not likewise occur in the pulmonary veins, and thus furnish us with our systemic embolus in a certain proportion of the cases. We must, surely, have recourse to the theory of an increased coagulability of the blood ; Almroth Wright, years ago, showed this to exist after influenza. If the germs associated with influenza and acute tonsillitis can cause jaundice (see Cases Nos. 1, 2 and 30) of hæmatogenous origin, it is not unlikely that a change in coagulability of the blood can be brought about by the same agency. The increased-coagulability theory will clear up many discrepancies ; it will account for the occurrence of the pulmonary, the systemic, and the so-called paradoxical embolisms. The word paradoxical, used in this connection, could thus be abolished. Is there invariable proof that, when more than one variety of embolism occurs in the same patient, they are both due to the same embolus or to emboli coming from the same source ? Is it not possible with such an increased coagulability of the blood that after the occurrence, say, of a pulmonary embolism a systemic embolism may be caused by a fresh embolus formed in another situation, and vice versa ?

I have no doubt that many of my readers will approach this essay in a spirit of scepticism, and may think that I have enthusiastically carried my conclusions farther than the evidence warrants. I am, however, very convinced of the truth of my statements. Unfortunately, the general practitioner has only his clinical instinct to guide him, as he rarely has a post-mortem to confirm or refute his suspicions. On the other hand, a majority of the cases do not die, and the interpretation of the symptoms and signs is always a matter of conjecture. Besides, the post-mortems at the large hospitals, though the most valuable help we have at present, form but a tithe of the fatal cases throughout the country ; much interesting material must, therefore, needs be missed. I find, however, that there is scientific proof of the occurrence of thrombosis and pulmonary embolism during influenza. Holtinger[14] in a recent interesting study entitled " Influenza and its complications in young children," described four cases of cerebral sinus thrombosis. It is interesting to me to note that in one of the cases (age $2\frac{1}{2}$ years) " embolisms were found in the left pulmonary arteries with an extensive infarct of the left lower lobe," and in another (age 19 months) " embolism of the right pulmonary artery." I have no doubt that most of us general practitioners have seen many similar cases, and have diagnosed them as " pneumonia." In addition, the Medical Registrar of one of the large London hospitals, Alan Moncrieff, in June, 1926, kindly wrote to inform me that he had seen "in the last six months three cases of pulmonary infarction all with autopsies." One of the cases followed a " white leg," the other two followed influenza, and were diagnosed as " influenzal pneumonia " during life; in both of them the embolus was derived from a thrombus in the pelvic veins.

I venture to think that an inquiry by all the large London and provincial hospitals into the incidence of all cases of embolism—without regard to whether they were medical or surgical, and with a note as to the time of their occurrence—would be productive of information of real value, and would probably bear out my theory of a " seasonal incidence of embolism," the seasons closely corresponding to the waves of influenza which are so frequently observed in general practice since the visitation of 1918.

*Prognosis.*—The prognosis in these cases of embolism, occurring as they do sometimes in healthy people, is by no means invariably unfavourable. While, of course, the occurrence of a minor pulmonary embolism makes one bear in mind the possibility of a subsequent fatal massive embolism, we are fortunately spared this in the great majority of the cases. Case No. 31 is an example, where a fatal embolism followed a non-fatal infarction. Generally speaking, the prognosis depends upon :—

1. *The previous history of the patient.*—Naturally one's prognosis is guarded in "cardiac" and arterio-sclerotic subjects, but, as will be seen by the histories of Cases Nos. 7 and 10, recovery may follow and the patients may live for years afterwards. Probably the embolus is a soft one in such cases.

2. *The size of the embolus.*—The larger the embolus the more likely is an immediately fatal result to ensue (in the pulmonary artery for instance), or irreparable damage to be done (in such an organ as the brain). With a smaller embolus, though alarming symptoms may at first be noted, re-establishment of the circulation may soon take place, and recovery may be astonishingly complete. It is in the lung that we can best observe the effect of different-sized emboli, as the symptoms and signs are less equivocal than in other regions.

3. *The nature of the embolus.*

(*a*) A septic embolus is likely to lead to pyæmia. Case No. 3 is a typical example of this kind. Fortunately, the septic embolus is rarer than the bland embolus.

(*b*) A soft or friable embolus may, almost immediately after causing alarming symptoms, break up into minute particles which pass on into the circulation, and recovery may be astonishingly complete. Only thus can one explain the amazing improvement seen in some of the cases in which embolism seems to be the only reasonable diagnosis. These "transient" embolisms, to use Osler's expression, are, I am sure, much commoner than we think.

The prognosis, anyway, in these cases, is much better than it used to be in the cases which one saw twelve or more years ago. At that time to get a "stroke" meant, in most cases, an inevitable paralysis to follow. Nowadays one can give the patient some cheer. The following most recent case of mine is an example of what I mean:—

**Case No. 32.**—J. H. P., a man aged 70, whose history was free from any previous serious complaint, and who was a remarkably active man for his years, felt all "one-sided" when doing a round of golf one day. He got back to his hotel, and woke up the next morning to discover that he had completely lost the use of his left upper limb. There was some weakness also in the lower limb, but he was able to move this. He came home, and when I saw him there was complete paralysis in his left arm. The heart action was rather feeble, but there was nothing else unfavourable. I told his wife and son—the latter a medical man—that there was quite a sporting chance of a great improvement. The stroke took place in the middle of September, 1926. A great improvement took place, and when I saw him last, on 18th December, I found him rolling his tobacco in an expert manner, with a strong grip and with almost normal movements in his arm. His son agreed with me that a thrombosis or embolism must have been the cause of the symptoms. Still well (1928). Had another attack, 1929, affecting speech.

Of the 32 numbered cases above 15 are now dead; Case No. 7 had lived for four years after the embolism. Of the deaths, 5 were due to pulmonary embolism, 7 to systemic embolism, whilst there was probably a mixed embolism in 3 of the cases. The list, however, is chosen at random, and contains a large proportion of fatal cases, as these are more liable to impress themselves on one's memory. As yet I have not had the time to make an exhaustive list of the cases.

*Treatment.*—This does not make a long story. The bold measure of opening the chest—as has been done on the continent—to remove a large pulmonary embolus is merely of academic interest to most of us. The removal of an embolus from the artery of a limb is a more feasible proposition, yet difficulties in technique have prevented this from being a very successful operation. On the theory of an increased coagulability of the blood in influenza and other infections the routine administration of the citrates in these diseases would seem a rational therapeutic measure. Is this the reason why the exhibition of potassium citrate is attended by such good results in cases of post-influenzal nephritis, or is it because it renders the urine alkaline ?

Are there any means by which we can prevent these embolic events ? I doubt it. Some surgeons, for example, say that we keep our patients too quiet after operation, and thus allow blood to stagnate—and clot—in their veins. I even have read somewhere that it is the placid type of patient who is liable to embolism. As a matter of fact, this is directly opposite to my own experience, that it is the restless, excitable patient who is liable to this complication. On the other hand, we are taught that if a patient has a thrombus anywhere he must be kept quiet for fear of dislodging the clot. How are we to choose the right path ? How are we to distinguish the pre-clot stage from the clot stage, especially as most of the clots that form are—in the pelvis—hidden and out of sight ? Some surgeons even attach importance to the method by which we tie our pedicles, as being a factor in the occurrence of post-operative embolism. One thing only can I say with any conviction. Infection is the most important factor in the ætiology of embolism, and thus no operations but the most urgent should be performed during the influenza seasons. Probably the only way in which we will, to any extent, prevent the occurrence of embolism will be by the prevention of influenza and other allied infections—a consummation, indeed, devoutly to be wished.

### CONCLUSIONS.

1. Influenza, like the other infectious diseases, is often the predisposing cause of all varieties of embolism.

2. Influenza is increasingly prevalent since the advent of the 1918 epidemics, and embolism is more commonly observed than formerly.

3. The embolism, though often occurring in the subjects of heart disease, occurs not infrequently in healthy and robust subjects.

4. The explanation which will account for the occurrence of embolism in healthy subjects and also for *all* varieties of embolism—pulmonary, systemic, and so-called paradoxical—is an increased coagulability of the blood.

5. The post-operative embolisms, whether pulmonary or systemic, are in many cases of a similar nature to the influenzal, and there is good reason for stating that they occur at the same seasons and that their numerical incidence varies directly with the prevalence of influenza.

6. Thrombosis in the pulmonary veins is probably an important factor in the production of systemic emboli which occur in patients with sound hearts.

### REFERENCES.

[1] Lockhart-Mummery, P., *Brit. Med. Jour.*, 1924, ii. 853.
[2] Lindsay, E. C., *Lancet*, 1925, i. 327.
[3] Wharton and Pierson, *Jour. Amer. Med. Assoc.*, 1919.
[4] Wyllie, W. G., and Moncrieff, A., *Lancet*, 1926, i. 128.
[5] Horder, Sir Thomas, *Lancet*, 1926, i. 749.
[6] Maingot, R., *Brit. Med. Jour.*, 1925, i. 1006.
[7] Gibson, A. G., *Lancet*, 1925, ii. 1270.
[8] Coombs, Carey, and Hadfield, Geoffrey, *Lancet*, 1926, i. 14.
[9] Jefferson, G., *Brit. Med. Jour.*, 1925, ii. 985.
[10] Featherstone, H. W., *Lancet*, 1925, i. 334.
[11] Gordon-Watson, Sir C., *Brit. Med. Jour.*, 1924, ii. 854.
[12] Whittingdale, J., *Lancet*, 1926, i. 575.
[13] Glynn, E., *Brit. Med. Jour.*, 1924, i. 323.
[14] Holtinger, A., *Monats. f. Kinderh.*, 1925, 30, 131.

## Figures and Tables

**Figure f1:**
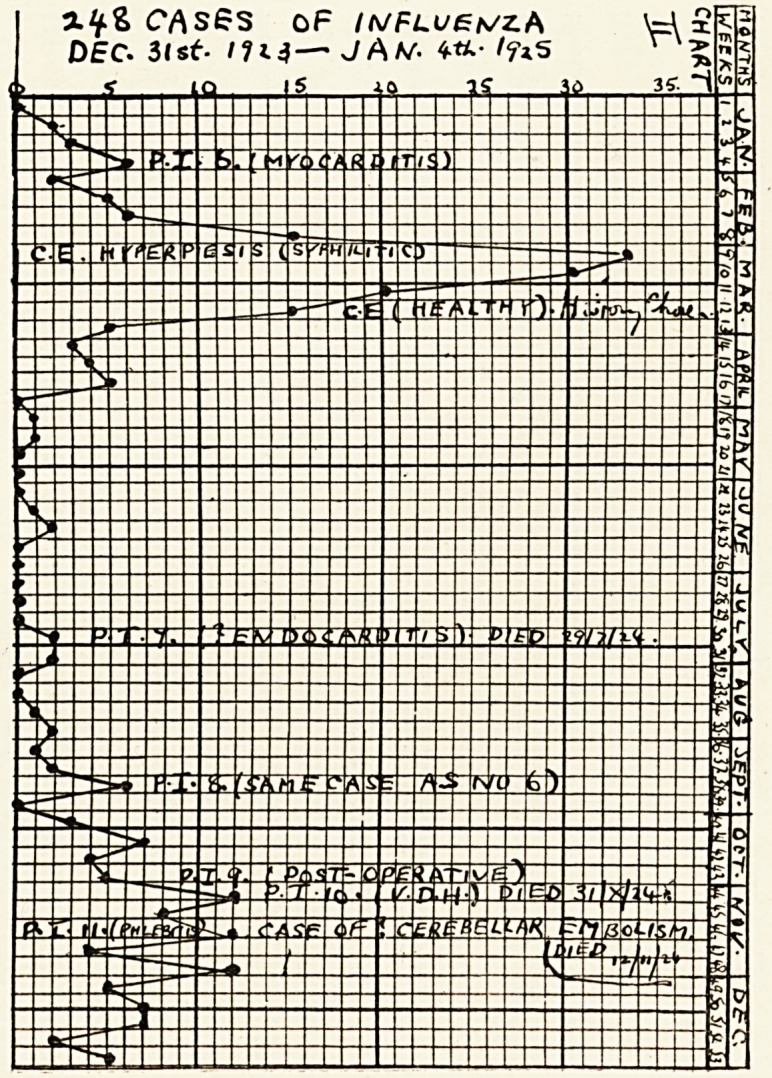


**Figure f2:**
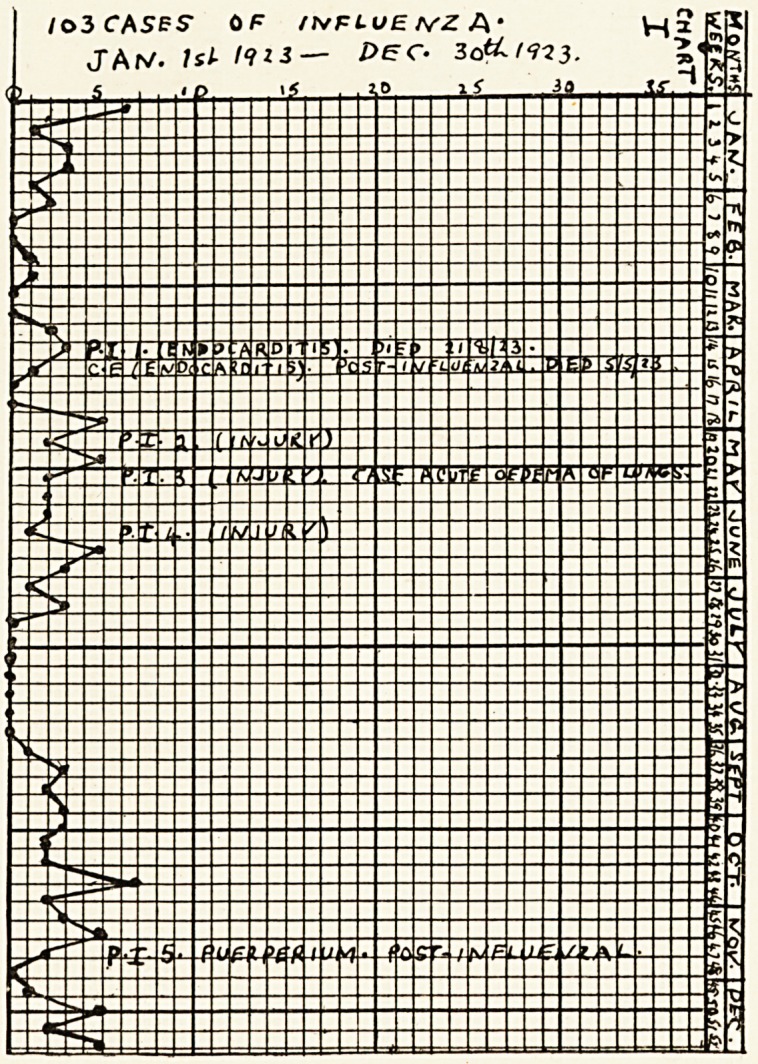


**Figure f3:**
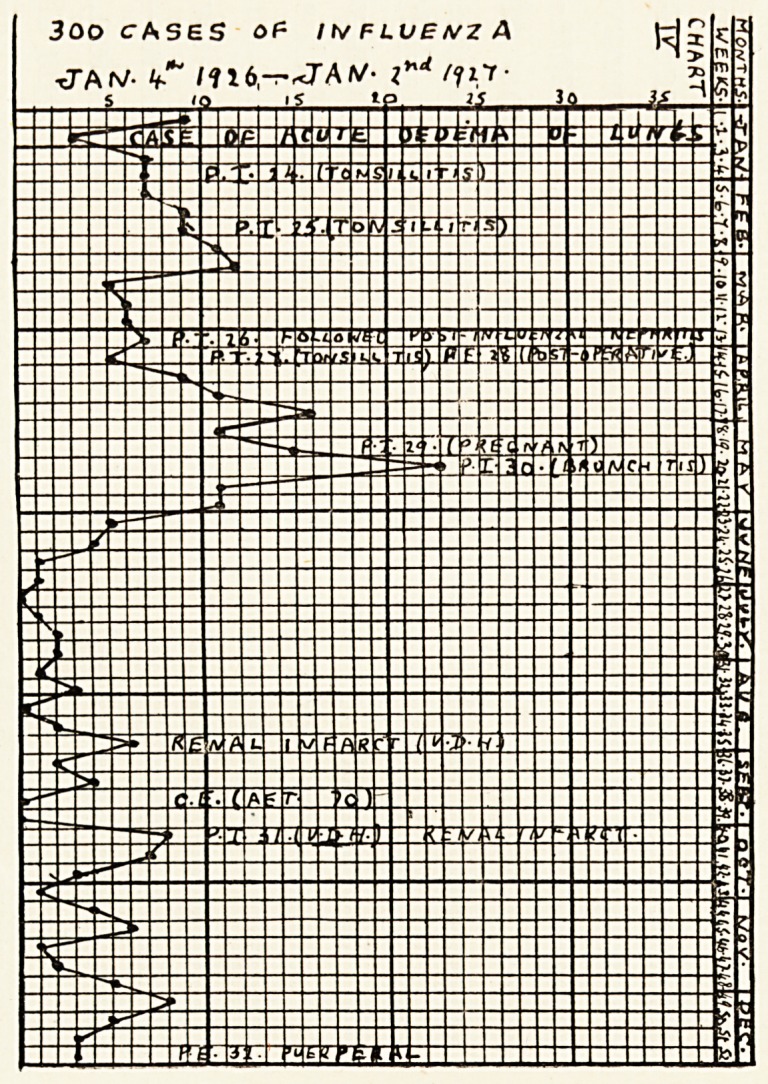


**Figure f4:**
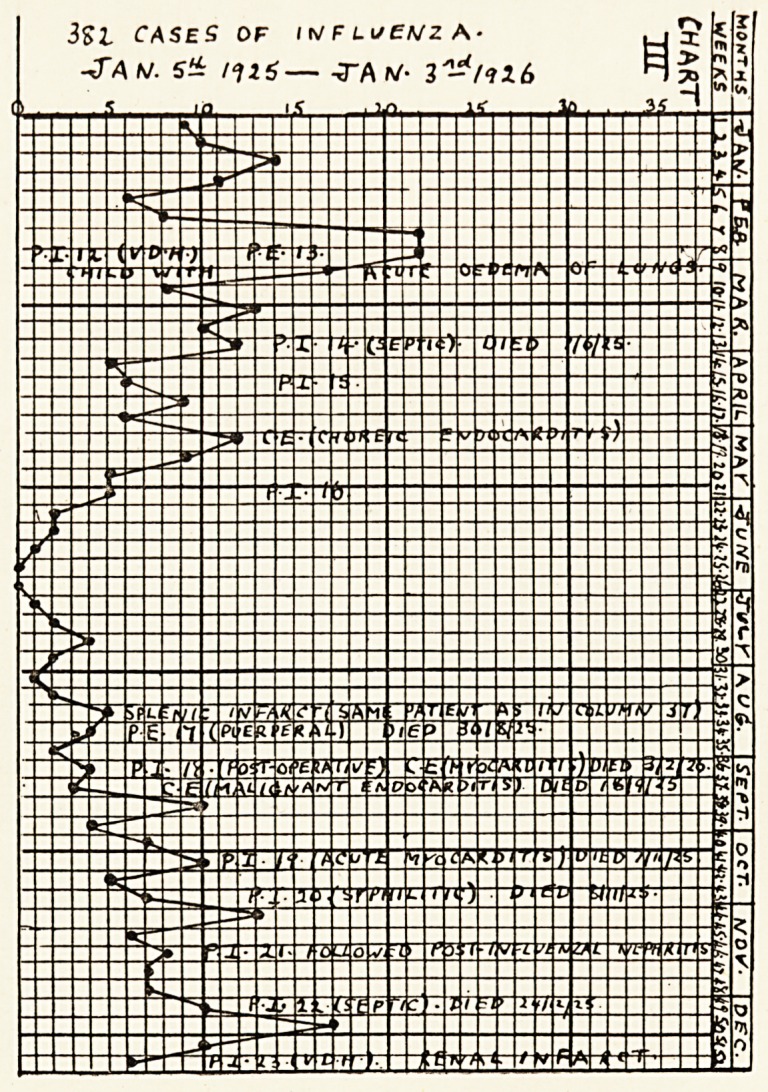


**Figure f5:**